# Oxidative stress impairs the calcification ability of human dental pulp cells

**DOI:** 10.1186/s12903-022-02467-w

**Published:** 2022-10-03

**Authors:** Satomi Shirawachi, Katsuhiro Takeda, Tomoya Naruse, Yohei Takahasi, Jun Nakanishi, Satoru Shindo, Hideki Shiba

**Affiliations:** 1 Department of Biological Endodontics, Graduate School of Biomedical and Health Sciences, Hiroshima University, 1-2-3 Kasumi, Minami-ku, Hiroshima, Hiroshima 734-8553 Japan; 2 Department of Oral Sciences and Translational Research, College of Dental Medicine, Nova Southeastern University, Fort Lauderdale, FL USA

**Keywords:** Oxidative stress, Human dental pulp cells, 2-aminoethyldiphenylborate

## Abstract

**Background:**

The relationship between internal root resorption and oxidative stress has not yet been reported. This study aimed to add molecular insight into internal root resorption. The present study was conducted to investigate the effect of hydrogen peroxide (H_2_O_2_) as an inducer of oxidative stress on the calcification ability of human dental pulp cells (hDPCs) and the involvement of inositol 1, 4, 5-trisphosphate (IP3).

**Material and methods:**

hDPCs (Lonza, Basel, Switzerland) were exposed to H_2_O_2_. Cell viability and reactive oxygen species (ROS) production were then evaluated. To investigate the effect of H_2_O_2_ on the calcification ability of hDPCs, real-time PCR for alkaline phosphatase (ALP) mRNA expression, ALP staining, and Alizarin red staining were performed. Data were compared with those of hDPCs pretreated with 2-aminoethyldiphenylborate (2-APB), which is an IP3 receptor inhibitor.

**Results:**

H_2_O_2_ at concentrations above 250 µM significantly reduced cell viability (*P* < 0.01). More ROS production occurred in 100 µM H_2_O_2_-treated hDPCs than in control cells (*P* < 0.01). 2-APB significantly decreased the production (*P* < 0.05). H_2_O_2_-treated hDPCs showed significant reductions in ALP mRNA expression (*P* < 0.01), ALP activity (*P* < 0.01), and mineralized nodule deposition compared with negative control cells (*P* < 0.01). 2-APB significantly inhibited these reductions (*P* < 0.01, *P* < 0.05 and *P* < 0.01, respectively). Data are representative of three independent experiments with three replicates for each treatment and values are expressed as means ± SD.

**Conclusion:**

To the best of our knowledge, this is the first study documenting the involvement of IP_3_ signaling in the calcification ability of human dental pulp cells impaired by H_2_O_2_.

## Introduction

Internal root resorption in permanent teeth is characterized by destruction of intraradicular dentin and dentinal tubules along the root canal wall [1]. It occurs in pathological conditions, including trauma, infections, or unknown causes [1, 2]. Concern and curiosity about resorption of dental structures are not recent. Numerous theories have been proposed as a possible cause of internal root resorption. One study reported that dental pulp cells have an innate ability to attenuate dentin resorption by inhibiting osteoclastogenesis [3]. Therefore, it is assumed that the damaged dental pulp cells are probably related to the onset of internal root resorption. However, the underlying pathology is not fully understood.

Dental pulp cells can differentiate into odontoblasts and generate a mineralizing matrix, particularly during reparative dentin formation associated with injury or disease [4, 5]. Odontoblasts, which are organized in a layer at the dentin-pulp interface, secrete type I collagen, osteocalcin (OCN), alkaline phosphatase (ALP), and other noncollagenous proteins [6]. The impairment of reparative dentin formation in permanent teeth may be a key factor implicated in internal root resorption.

Oxidative stress is a damaging response and refers to excessive intracellular levels of reactive oxygen species (ROS). Hydrogen peroxide (H_2_O_2_) is one of the major ROS. At low physiological levels in the nanomolar range, H_2_O_2_ is the major agent signaling through specific protein targets that engage in metabolic regulation and stress responses to support cellular adaptation to a changing environment and stress. Many previous studies reported the effects of oxidative stress on pulp cells [7–9]. One study showed that the oxidative stress of human dental pulp cells mediated by H_2_O_2_ promotes reduction of odontoblastic capability [8]. On the other hand, Matsu et al. [9] found an increase in osteopontin (OPN) and osteocalcin (OCN) in H_2_O_2_-treated-human dental pulp cells. Further investigations seem to be needed to clarify the mechanism. In addition, the relationship between internal root resorption and oxidative stress has not yet been reported. This study focused on H_2_O_2_ as an inducer of oxidative stress.

2-Aminoethyldiphenylborate (2-APB) was originally described as a membrane-permeant inhibitor of the inositol 1, 4, 5-trisphosphate (IP_3_) receptor, and it was also used as one of the store-operated Ca^2+^ entry (SOCE) inhibitors [10]. Yamamura et al. [11] reported that oxidative stress reduced SOCE, and the decrease was recovered by 2-APB in brain capillary endothelial cells.

The present study was conducted to add molecular insight into root canal resorption. In the current study, it was demonstrated for the first time that IP_3_ signaling is involved in the calcification ability of H_2_O_2_-treated human dental pulp cells. A possible mechanism to explain the influence of the oxidative stress caused by H_2_O_2_ on the calcification ability of human dental pulp cells is presented. The present study may provide a clue to help clarify a mechanism of onset of internal root resorption.

## Materials and methods

### Reagents

Hydrogen peroxide (H_2_O_2_) and 2-aminoethoxydiphenylborane (2-APB) were purchased from FUJIFILM Wako Pure Chemicals Corporation (Osaka, Japan) and Bio-Techne Corporation (Minneapolis, MN, USA), respectively.

### Cell culture

Human dental pulp cells (hDPCs) were obtained from Lonza (Basel, Switzerland). Cells were cultured in growth medium (GM) (Eagle’s Minimum Essential Medium, ALPHA modification (MEM-α)) supplemented with 10% heat-inactivated FBS (Fetal Bovine Serum; Sigma-Aldrich, MO, USA), 100 IU/mL penicillin (FUJIFILM Wako Pure Chemicals Corp.), and 100 µg/mL streptomycin (FUJIFILM Wako Pure Chemicals Corp.) (medium A). The cells were incubated in a humidified atmosphere of 5% CO_2_ and 95% air at 37 °C. Cell cultures between the sixth and ninth passages were used in this study.

To differentiate into odontoblast-like cells, the cells were cultured in osteo-odontogenic medium (OM), MEM-α supplemented with 2.5% heat-inactivated FBS (Sigma-Aldrich), 100 IU/mL penicillin (FUJIFILM Wako Pure Chemicals Corp.), 100 µg/mL streptomycin (FUJIFILM Wako Pure Chemicals Corp.), 10 mM β-glycerophosphate (Tokyo Chemical Industry Co., Ltd., Tokyo, Japan), and 50 mg/mL ascorbic acid (FUJIFILM Wako Pure Chemicals Corp.).

### WST assay

Cell viability was assessed by the WST assay using the Cell Counting Kit-8 according to the manufacturer’s protocol (CCK-8; Dojindo Laboratories, Kumamoto, Japan). Briefly, cells were plated at a density of 5.0 × 10^3^ cells/well in 96-well plates and maintained in 0.1 mL of medium A. After 24-h incubation, culture medium was replaced to serum-free medium with H_2_O_2_ at concentrations of 0, 50, 100, 250, 500, and 1000 µM (final concentration) and incubated for 24 h in a humidified atmosphere of 5% CO_2_ and 95% air at 37 °C. Then, CCK-8 solution was added, and the cells were incubated for another hour. Absorbance at 450 nm was then measured with the reference wavelength at 620 nm using a microplate reader (Multiskan™ FC; Thermo Fisher Scientific, CA, USA). Data are representative of three independent experiments with three replicates for each treatment.

### Intracellular reactive oxygen species measurements

Measurement of intracellular ROS was performed by dichlorodihydrofluorescein diacetate (DCFH-DA) (ROS Assay Kit-Highly Sensitive DCFH-DA-; Dojindo Laboratories). Cells were seeded at a density of 1.0 × 10^4^ cells in 6-well plates and incubated overnight in medium A. The medium was replaced to serum-free medium with 10 µM 2-APB or PBS for control cells. One hour later, the cells were stimulated with 100 µM H_2_O_2_ for 2 h in a humidified atmosphere of 5% CO_2_ and 95% air at 37 °C. DCFH-DA solution was added after 3-h incubation, and the cells were then incubated for another 30 min. Fluorescence signals were observed using an all-in-one fluorescence microscope (BZ-X700; Keyence, Tokyo, Japan). Data are representative of three independent experiments with three replicates for each treatment.

### ALP staining

To evaluate alkaline phosphatase activity, the cells were stained by BCIP-NBT solution (SIGMA FAST™BCIP®/NBT; Sigma-Aldrich). hDPCs were seeded into 48-well plates at a density of 1.0 × 10^4^ cells/well in medium A. After the cells reached 70–80% confluency, culture medium was replaced to fresh MEM-α supplemented with 2.5% FBS (Sigma-Aldrich), 100 IU/mL penicillin (FUJIFILM Wako Pure Chemicals Corp.), and 100 µg/mL streptomycin (FUJIFILM Wako Pure Chemicals Corp.) (medium B). The cells were treated with 10 µM 2-APB or PBS for control cells. One hour later, cells were stimulated with 100 µM H_2_O_2_ for 6 h. Then, medium was replaced to OM and cultured for 7 days. The medium was changed on the third day. After incubation, the cells were rinsed with PBS, fixed with 10% neutral buffered formalin, and stained with BCIP-NBT solution. The stained area was quantified by Image J software (National Institutes of Health, Bethesda, MD, USA). To briefly explain the quantification method, images were converted to RGB stack, and “blue slice” images were picked up (Fig. 2A(b)). The threshold of the blue slice images was adjusted, and a limited threshold area was highlighted in red, as shown in Fig. 2A(c). Thereafter, the % stained area was automatically measured by the software. Data are representative of three independent experiments with three replicates for each treatment.

### Alizarin red staining

hDPCs were seeded into 6-well plates at a density of 1.0 × 10^5^ cells/well and cultured in medium A. After the cells reached 70–80% confluency, the culture medium was replaced to medium B and treated with 10 µM 2-APB or PBS for control cells. One hour later, cells were stimulated with 100 µM H_2_O_2_ for 6 h. The medium was then replaced to OM and cultured for 28 days. The medium was changed every 3 days. To evaluate calcium deposition, Alizarin red S Solution (PG research, Tokyo, Japan) was used after fixation with 10% neutral buffered formalin. The images were captured by an all-in-one fluorescence microscope (BZ-X700; Keyence), and the stained area was quantified by Image J software (National Institutes of Health). Data are representative of three independent experiments with three replicates for each treatment.

### Isolation of total RNA and reverse transcription

hDPCs were seeded into 6-well plates at a density of 1.0 × 10^5^ cells/well and cultured in medium A until subconfluent. The cells were treated with 10 µM 2-APB or PBS for control cells. One hour later, the cells were stimulated with 100 µM H_2_O_2_ for 6 h. The cells were replaced to OM and cultured for 7 days. The medium was changed on the third day. Total RNA from each culture was extracted using RNA iso plus (Takara Bio Inc., Shiga, Japan) according to the manufacturer’s protocol. cDNA was synthesized from 250 ng of total RNA in a final volume of 10 µL using the Rever Tra Ace qPCR RT Master Mix with gDNA remover kit (Toyobo Co., Ltd, Osaka, Japan) with a thermal cycler (Veriti™ 96-Well Thermal Cycler; Life Technologies, CA, USA).

### Real-time PCR

Two-step qPCR was performed with the StepOnePlus™ Real-Time PCR System (Thermo Fisher Scientific, CA, USA) using THUNDERBIRD® Next SYBR® qPCR Mix (Toyobo Co., Ltd, Osaka, Japan). The cycling protocol was as follows: DNA polymerase activation at 95 °C for 30 s, followed by denaturation at 95 °C for 5 s and annealing/extension at 60 °C for 10 s, for 40 cycles in fast mode. Gene expression was normalized to that of *Gapdh* mRNA in the same samples, using the 2^−ΔΔCt^ method. The sequences of relevant primers were as follows: *Gapdh*: forward 5′-AACGTGTCAGTGGTGGACCTG-3′; reverse 5′-AGTGGGTGTCGCTGTTGAAGT-3′; *ALP*: forward 5′-CGCCTACCAGCTCATGCATAAC-3′; reverse 5′-GTCAATTCTGCCTCCTTCCACC-3′. Data are representative of three independent experiments with three replicates for each treatment.

### Statistical analysis

Each result is presented as a mean ± standard error of the mean. All experiments were performed in at least triplicate. Comparisons between two groups were evaluated by an unpaired two-tailed Student’s *t-*test. For comparisons of more than two groups, one-way ANOVA was followed by Tukey’s test for multiple comparisons. Significance was set at *p* < 0.05.

## Results

**Fig. 1:**
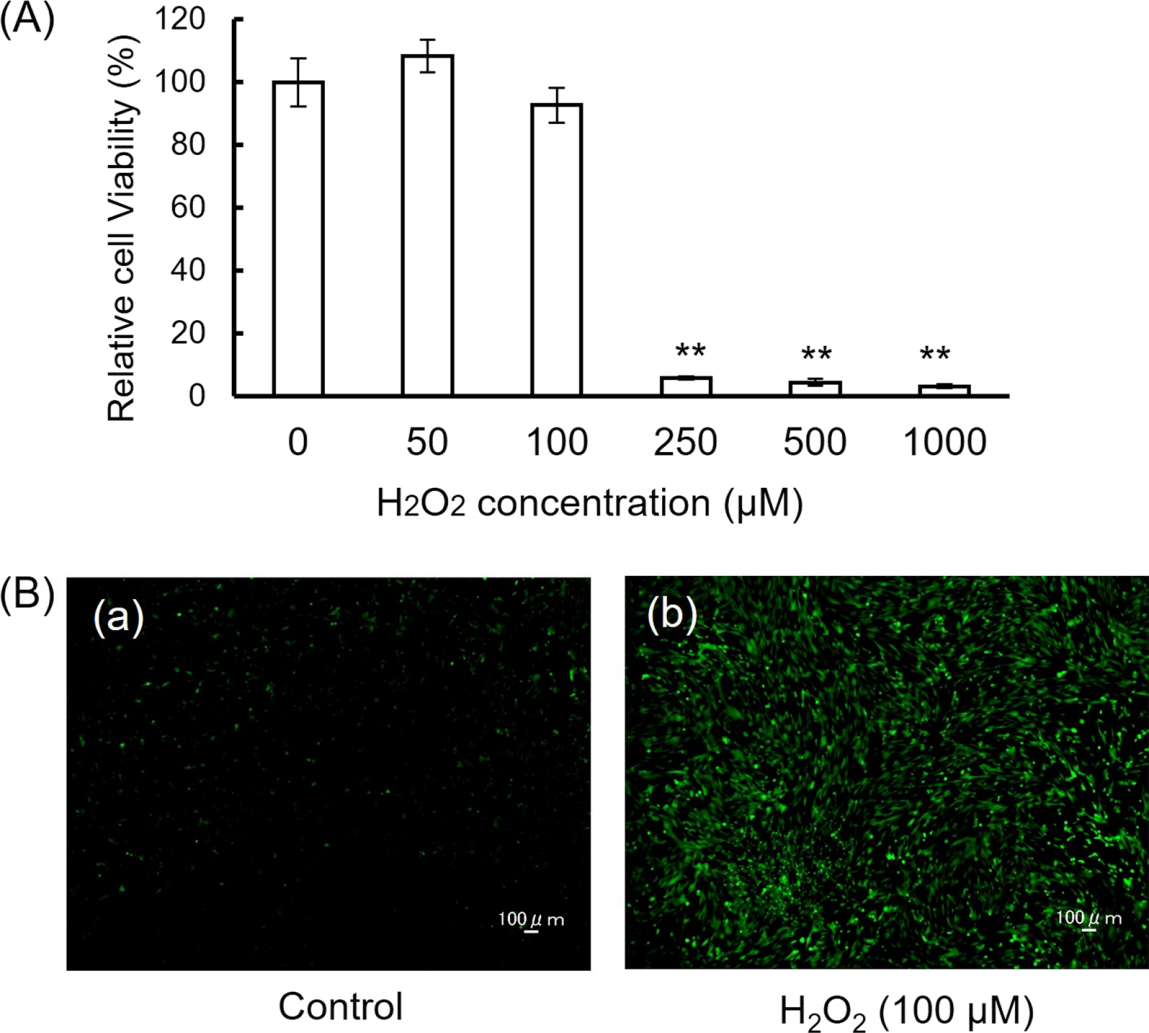
Cell viability and ROS production of H_2_O_2_-treated human dental pulp cells. **A** Cell viability is assessed by the WST assay using Cell Counting Kit-8. hDPCs were treated with H_2_O_2_ at concentrations of 50, 100, 250, 500, and 1000 µM for 24 h. Data are presented as means ± SD of three independent experiments. **P < 0.01, one-way ANOVA with Tukey’s test. **B** ROS production in hDPCs. Fluorescence signals were detected with a confocal microscope. (a) Control, (b) H_2_O_2_ 100 μM. Bars: 100 μm

hDPCs underwent severe cell death after high-dose H_2_O_2_ (250, 500 and 1000 μM) treatment for 24 h as determined by the WST assay (Fig. 1A). H_2_O_2_ at 50 and 100 µM did not decrease cell viability. ROS were increased in 100 μM H_2_O_2_-treated hDPCs (Fig. 1B). Therefore, in the present study, H_2_O_2_ at 100 μM, which is enough to promote ROS production, was used.

**Fig. 2:**
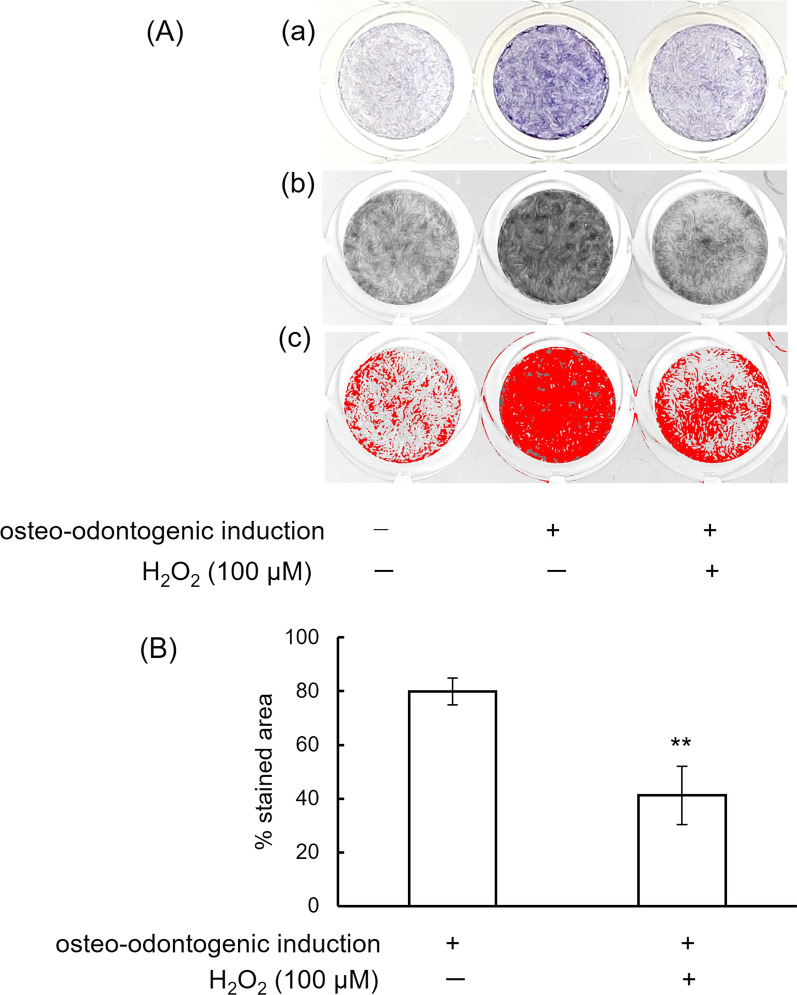
H_2_O_2_ attenuates ALP activity of hDPCs. **A**(a) A macroscopic view of wells showing hDPCs cultured in growth medium, hDPCs cultured in osteo-odontogenic medium, and hDPCs treated with 100 μM H_2_O_2_ in osteo-odontogenic medium. (b) **A**(a) panels were converted to RGB stack, and “blue slices” were picked up. (c) The thresholds of the images of **A**(b) were adjusted, and a limited threshold area is highlighted in red. **B** The stained area was quantified using Image J software on digitized photomicrographs (**A**(c)) captured by a Windows-based computer. Values are expressed as means ± SD of three independent experiments. **P < 0.01, Student’s *t*-test

To determine whether H_2_O_2_ has an effect on the calcification ability of hDPCs in the present experimental system, the effects of H_2_O_2_ on hDPCs cultured in osteo-inductive medium were tested via ALP activity. After osteogenic differentiation for 7 days, H_2_O_2_ at 100 μM significantly decreased ALP activity in hDPCs (*P* < 0.01) (Fig. 2A, B).

**Fig. 3.**
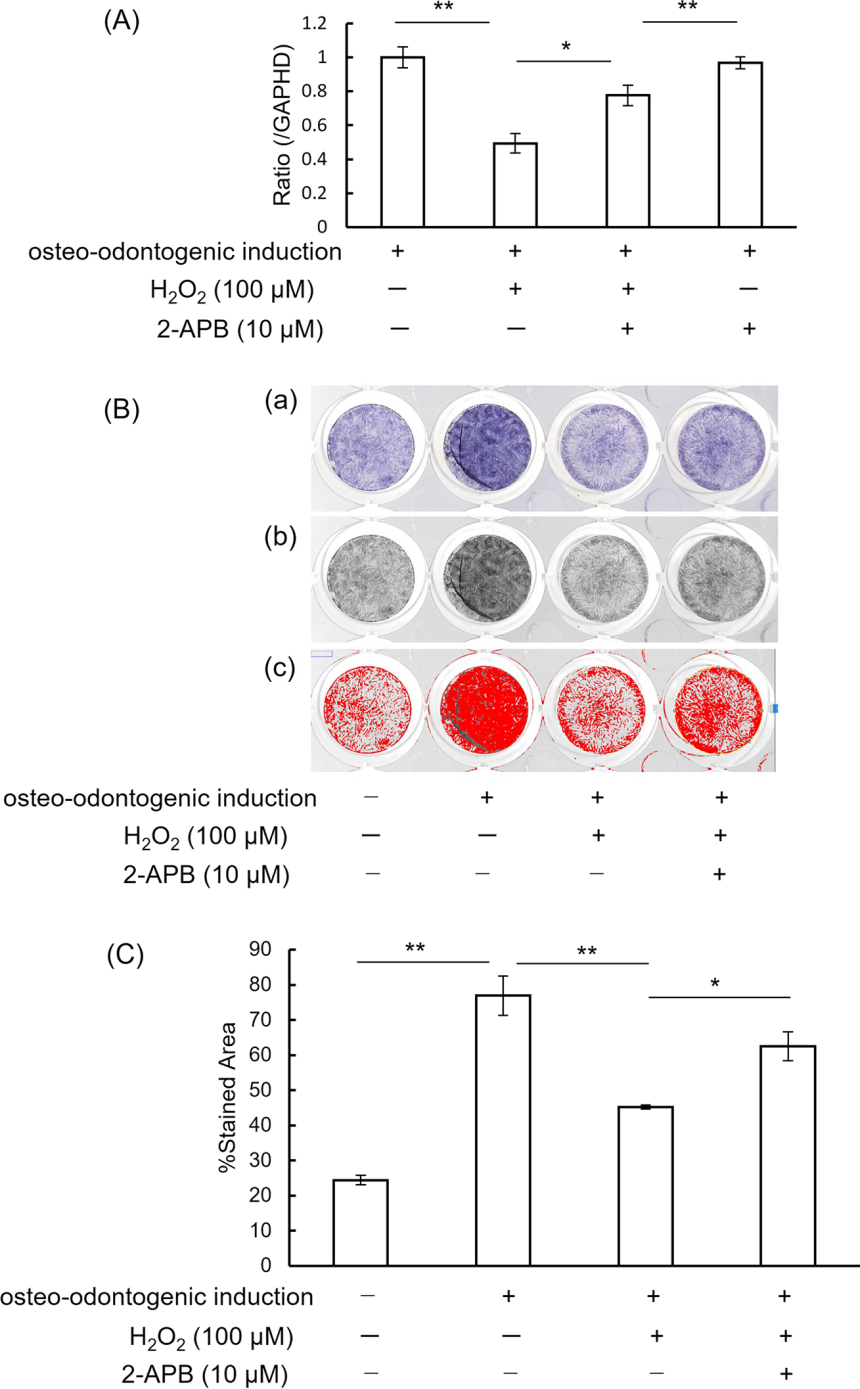
2-APB reverses H_2_O_2_-decreased ALP mRNA expression and ALP activity of hDPCs. **A** ALP mRNA was determined by real-time PCR. Graphs show the ratio of ALP mRNA to GAPDH mRNA. Values represent means ± SD of three cultures. *p < 0.05, **p < 0.01: differs significantly from the control. **B**(a) A macroscopic view of wells showing hDPCs cultured in growth medium, hDPCs cultured in osteo-odontogenic medium, hDPCs treated with 100 μM H_2_O_2_ in osteo-odontogenic medium, and hDPCs treated with 10 μM 2-APB before the addition of 100 μM H_2_O_2_ in osteo-odontogenic medium. (b) **B**(a) panels have been converted to RGB stack, and “blue slice” images were picked up. (c) The thresholds of **B**(b) images were adjusted, and a limited threshold area is highlighted in red. **C** The stained area was quantified using the software NIH Image J^®^ on digitized photomicrographs (**B**(c)) captured by a Windows-based computer. Data are shown as means ± SD of three independent experiments. *P < 0.05, **P < 0.01, one-way ANOVA with Tukey’s test

Real-time PCR was used to determine the effect of H_2_O_2_ addition on the mRNA expression of ALP. H_2_O_2_ significantly decreased the ALP mRNA levels compared with control cells (*P* < 0.01). 2-APB reversed the decrease significantly (*P* < 0.05) (Fig. 3A).

The percentages of the BCIP-NBT stained area of the control group, positive control group (cultured in osteo-inductive medium), H_2_O_2_ group and H_2_O_2_/2-APB treated group were 24.4 ± 1.3%, 76.9 ± 5.9%, 45.2 ± 0.6%, and 62.6 ± 4.1%, respectively. H_2_O_2_ down-regulated ALP activity compared with positive control cells (cultured in osteo-inductive medium). 2-APB significantly rescued the decrease of ALP activity in H_2_O_2_-treated cells (*P* < 0.01) (Fig. 3B, C).

**Fig. 4:**
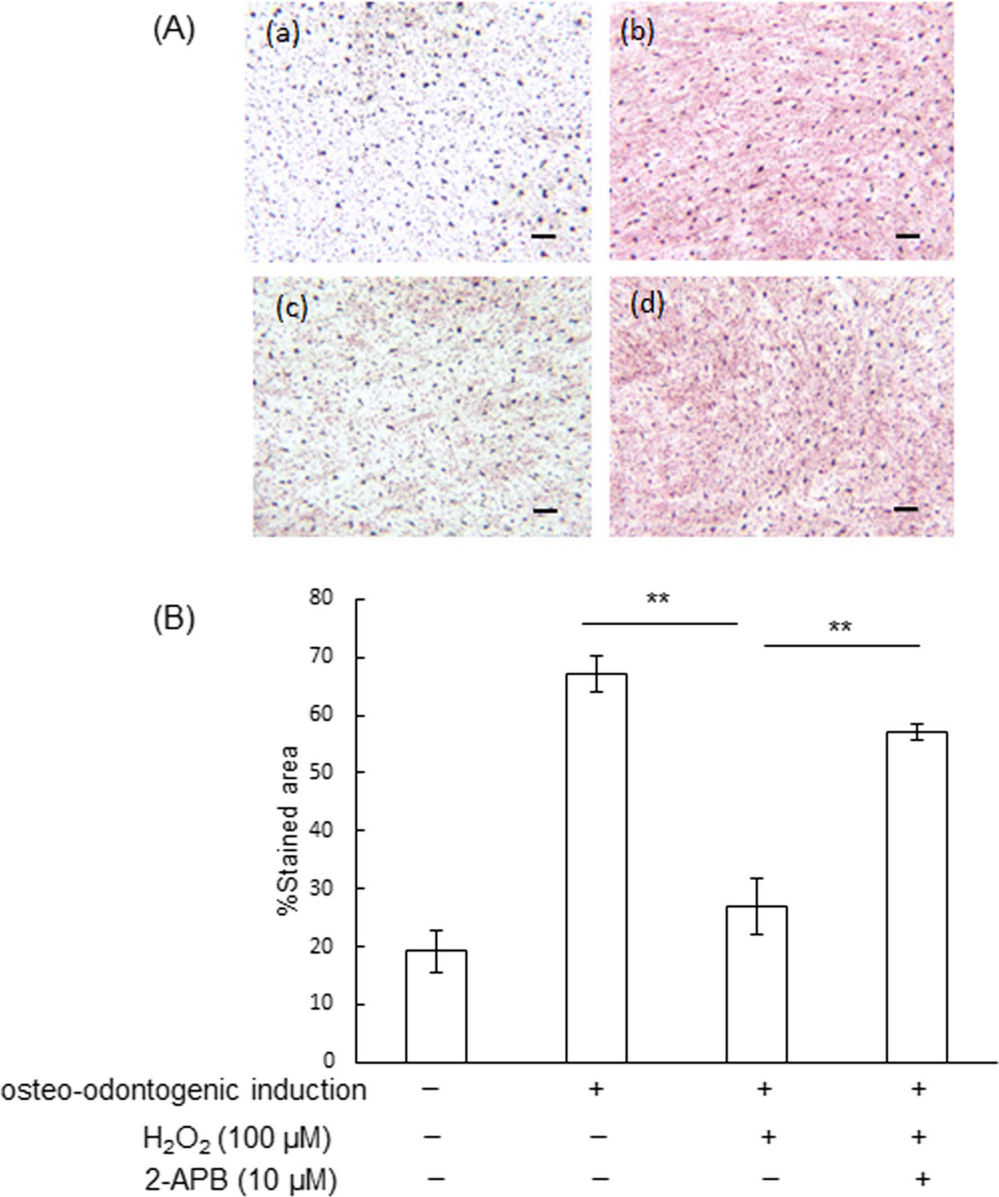
Assessment of hDPC calcification with Alizarin red staining. **A** Panels show hDPCs cultured in growth medium (a), hDPCs cultured in osteo-odontogenic medium (b), hDPCs treated with 100 μM H_2_O_2_ in osteo-odontogenic medium (c) and hDPCs treated with 10 μM 2-APB before the addition of 100 μM H_2_O_2_ in osteo-odontogenic medium (d). Bars: 100 μm. **B** The amount of calcium was quantified using Image J software on digitized photomicrographs captured by a Windows-based computer. Data are presented as means ± SD of three independent experiments. **P < 0.01, one-way ANOVA with Tukey’s test

The percentages of the Alizarin red-stained area of the control group, positive control group (cultured in osteo-inductive medium), H_2_O_2_ group, and H_2_O_2_/2-APB treated group were 19.1 ± 3.6%, 67.1 ± 3.2%, 26.9 ± 4.8%, and 57.1 ± 1.5%, respectively. Alizarin red staining showed that 2-APB significantly increased H_2_O_2_-decreased hDPC calcification (*P* < 0.01) (Fig. 4A, B).

**Fig. 5.**
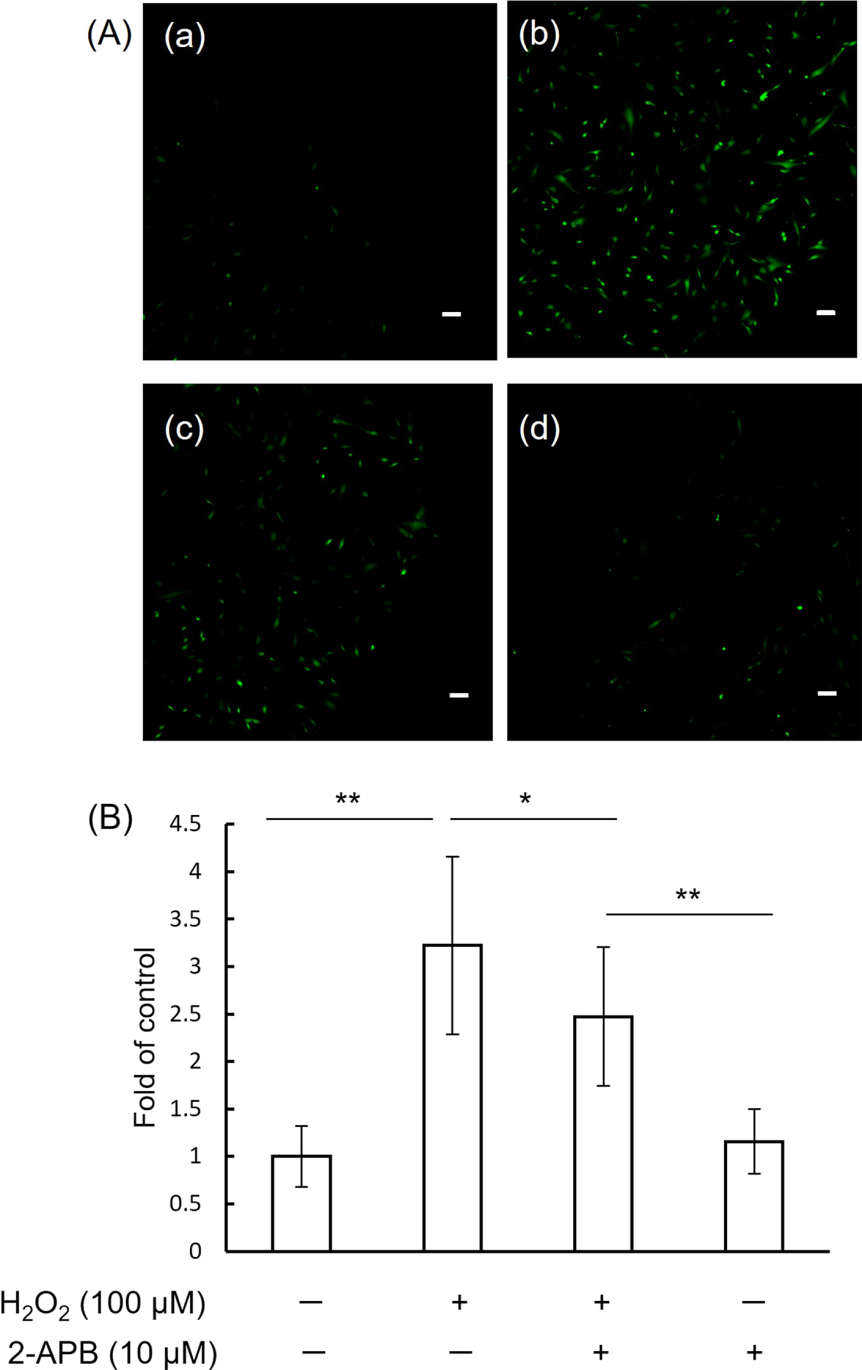
2-APB inhibits the ROS production induced by H_2_O_2_ in hDPCs. **A** ROS production in hDPCs. Fluorescence signals were detected with a confocal microscope. (a) control, (b) H_2_O_2_ 100 μM, (c) H_2_O_2_ 100 μM + 2-APB 10 μM, (d) 2-APB 10 μM. Bars: 100 μm. **B** The graph shows quantitative results of ROS production from three independent experiments. Per well, three pictures were taken at random. FITC fluorescence intensity from an area of 2 mm square from each picture was analyzed using Image J software on digitized photomicrographs captured by a Windows-based computer. Data represent means ± SD. *P < 0.05, **P < 0.01, one-way ANOVA with Tukey’s test

ROS were increased in 100 μM H_2_O_2_-treated hDPCs (Fig. 5A(a, b). 10 µM 2-APB inhibited the ROS production induced by 100 μM H_2_O_2_ (Fig. 5A(c)). 10 µM 2-APB did not affect the ROS production(Fig. 5A(d)). FITC fluorescence intensity of the H_2_O_2_/2-APB treated group was significantly decreased compared to that of the H_2_O_2_-treated group (*P* < 0.05) (Fig. 5B).

## Discussion

H_2_O_2_ promotes ROS production in various cells, such as human periodontal ligament cells, human retinal epithelial cells, and vascular endothelial cells [12–14]. H_2_O_2_ can induce oxidative destruction of organs and tissues, associated with other inflammatory responses when overexpressed in cells [15]. The effect of H_2_O_2_ in a living system is dependent on the type of cell, its concentration, its physiological state, and duration of exposure [16, 17]. Deng et al. [18] reported that, like other ROS molecules, a high concentration of H_2_O_2_ is cytotoxic to cells. In the present study, high-dose H_2_O_2_ (250, 500 and 1000 μM) significantly reduced the viability of hDPCs. Loss of cell viability is one characteristic of apoptotic cells. Therefore, in the present study, to avoid the influence of apoptosis, H_2_O_2_ at 100 μM, which is enough to promote ROS production without inducing apoptosis, was used.

Alkaline phosphatase (ALP), involved with the initial phase of dentin matrix biomineralization, promotes dephosphorylation of extracellular matrix proteins, providing inorganic phosphate [19]. In the present study, H_2_O_2_ decreased ALP activity in hDPCs cultured in osteo-inductive medium. These data are in agreement with those from previous studies [8, 20]. Correlated with ALP activity, the present study also showed decreased ALP mRNA expression by H_2_O_2_ in hDPCs.

2-APB has been described as a membrane-permeant inhibitor of the IP_3_ receptor, and the inhibition of SOCE by 2-APB was taken as evidence for IP_3_ receptor activation of calcium release-activated Ca^2+^ channels [21]. It has been reported that the increase in intercellular Ca^2+^ concentration regulates cell functions, such as proliferation, differentiation, and migration [22]. A previous study reported that oxidative stress reduced SOCE, and the decrease was reversed by 2-APB in brain capillary endothelial cells [11]. In the present study, the H_2_O_2_-decreased human dental pulp cell calcification ability was reversed by 2-APB. In addition, an important criterion for the characterization of odontoblastic cells is their ability to mineralize the collagenous matrix they secrete. In the present study, 2-APB rescued the H_2_O_2_-decreased amounts of mineralization of the extracellular matrix, judged by Alizarin red staining. Contrary to the present findings, it has been reported that vascular smooth muscle cell calcification was reduced by 2-APB [23, 24]. 2-APB is also used as an inhibitor of transient receptor potential channels (TRPCs), in which Ca^2+^ entry was inhibited directly, rather than as a result of the inhibition of IP_3_ [25]. Further experiments are needed to clarify the detailed mechanism underlying the relationships between IP_3_, SOCE, TRPC and the calcification ability of human dental pulp cells.

It has been reported that 2-APB reduced ROS production in neutrophils and prevented ROS-induced cardiomyocyte death [26, 27]. The present study showed for the first time that 2-APB reduces ROS production in hDPCs. Therefore, administration of 2-APB may represent a promising therapeutic strategy for the treatment of ROS-related endodontic disease. Further experiments beyond the scope of this paper are required to determine the relationship between ROS and endodontic disease.

In summary, H_2_O_2_ decreased the calcification ability of human dental pulp cells, and the reduction was reversed by 2-APB. To the best of our knowledge, this is the first study documenting the involvement of IP_3_ signaling in the calcification ability of human dental pulp cells. The results of the present study add molecular insight into internal root resorption and may provide a clue to the development of a new therapeutic agent for such resorption in endodontic therapy.

## Data Availability

The datasets generated during or analysed during the current study are available from the corresponding author on reasonable request.

## Abbreviations

hDPCs: Human dental pulp cells
H_2_O_2_: Hydrogen peroxide
ROS: Reactive oxygen species
2-APB: 2-Aminoethyldiphenylborate

## References

[1] Gayathri P, Pandey RK, Jain E (2014). Management of internal resorption of central incisor using hybrid technique. BMJ Case Rep.

[2] Kandalgaonkar SD, Gharat LA, Tupsakhare SD, Gabhane MH (2013). Invasive cervical resorption: a review. J Int Oral Health.

[3] Zheng Y, Chen M, He L, Marão HF, Sun DM, Zhou J, Kim SG, Song S, Wang SL, Mao JJ (2015). Mesenchymal dental pulp cells attenuate dentin resorption in homeostasis. J Dent Res.

[4] Couble ML, Farges JC, Bleicher F, Perrat-Mabillon B, Boudeulle M, Magloire H (2000). Odontoblast differentiation of human dental pulp cells in explant cultures. Calcif Tissue Int.

[5] Suzuki T, Nomura S, Maeda T, Ohshima H (2004). An immunocytochemical study of pulpal responses to cavity preparation by laser ablation in rat molars by using antibodies to heat shock protein (Hsp) 25 and class II MHC antigen. Cell Tissue Res.

[6] Butler WT, Ritchie H (1995). The nature and functional significance of dentin extracellular matrix proteins. Int J Dev Biol.

[7] Lee YH, Kang YM, Heo MJ, Kim GE, Bhattarai G, Lee NH, Yu MK, Yi HK (2013). The survival role of peroxisome proliferator-activated receptor gamma induces odontoblast differentiation against oxidative stress in human dental pulp cells. J Endod.

[8] Soares DG, Gonçalves Basso F, Hebling J, de Souza Costa CA (2015). Effect of hydrogen-peroxide-mediated oxidative stress on human dental pulp cells. J Dent.

[9] Matsui S, Takahashi C, Tsujimoto Y, Matsushima K (2009). Stimulatory effects of low-concentration reactive oxygen species on calcification ability of human dental pulp cells. J Endod.

[10] DeHaven WI, Smyth JT, Boyles RR, Bird GS, Putney JW (2008). Complex actions of 2-aminoethyldiphenyl borate on store-operated calcium entry. J Biol Chem.

[11] Yamamura H, Suzuki Y, Asai K, Imaizumi Y (2020). Oxidative stress facilitates cell death by inhibiting Orai1-mediated Ca(2+) entry in brain capillary endothelial cells. Biochem Biophys Res Commun.

[12] Chae SY, Park SY, Park G (2018). Lutein protects human retinal pigment epithelial cells from oxidative stress-induced cellular senescence. Mol Med Rep.

[13] Ruan Y, Wu S, Zhang L, Chen G, Lai W (2014). Retarding the senescence of human vascular endothelial cells induced by hydrogen peroxide: effects of 17beta-estradiol (E2) mediated mitochondria protection. Biogerontology.

[14] Kuang Y, Hu B, Feng G, Xiang M, Deng Y, Tan M, Li J, Song J (2020). Metformin prevents against oxidative stress-induced senescence in human periodontal ligament cells. Biogerontology.

[15] Park H, Kim S, Song Y, Seung K, Hong D, Khang G, Lee D (2010). Antioxidant and anti-inflammatory activities of hydroxybenzyl alcohol releasing biodegradable polyoxalate nanoparticles. Biomacromol.

[16] Lennicke C, Rahn J, Lichtenfels R, Wessjohann LA, Seliger B (2015). Hydrogen peroxide: production, fate and role in redox signaling of tumor cells. Cell Commun Signal.

[17] Schieber M, Chandel NS (2014). ROS function in redox signaling and oxidative stress. Curr Biol.

[18] Deng Q, Liu Q, Zhang H, Fan W, Li J, Kang J, He H, Huang F (2019). Melatonin enhances hydrogen peroxide-induced apoptosis in human dental pulp cells. J Dent Sci.

[19] Goldberg M, Kulkarni AB, Young M, Boskey A (2011). Dentin: structure, composition and mineralization. Front Biosci (Elite Ed).

[20] de Oliveira Duque CC, Soares DG, Basso FG, Hebling J, de Souza Costa CA (2017). Influence of enamel/dentin thickness on the toxic and esthetic effects of experimental in-office bleaching protocols. Clin Oral Investig.

[21] Ma HT, Patterson RL, van Rossum DB, Birnbaumer L, Mikoshiba K, Gill DL (2000). Requirement of the inositol trisphosphate receptor for activation of store-operated Ca^2+^ channels. Science.

[22] Nilius B, Droogmans G (2001). Ion channels and their functional role in vascular endothelium. Physiol Rev.

[23] Montezano AC, Zimmerman D, Yusuf H, Burger D, Chignalia AZ, Wadhera V, van Leeuwen FN, Touyz RM (2010). Vascular smooth muscle cell differentiation to an osteogenic phenotype involves TRPM7 modulation by magnesium. Hypertension.

[24] Lee CT, Ng HY, Kuo WH, Tain YL, Leung FF, Lee YT (2020). The role of TRPM7 in vascular calcification: comparison between phosphate and uremic toxin. Life Sci.

[25] Bencze M, Behuliak M, Vavřínová A, Zicha J (2015). Broad-range TRP channel inhibitors (2-APB, flufenamic acid, SKF-96365) affect differently contraction of resistance and conduit femoral arteries of rat. Eur J Pharmacol.

[26] Conejeros I, Jara E, Carretta MD, Alarcón P, Hidalgo MA, Burgos RA (2012). 2-Aminoethoxydiphenyl borate (2-APB) reduces respiratory burst, MMP-9 release and CD11b expression, and increases l-selectin shedding in bovine neutrophils. Res Vet Sci.

[27] Morihara H, Obana M, Tanaka S, Kawakatsu I, Tsuchiyama D, Mori S, Suizu H, Ishida A, Kimura R, Tsuchimochi I, Maeda M, Yoshimitsu T, Fujio Y, Nakayama H (2017). 2-aminoethoxydiphenyl borate provides an anti-oxidative effect and mediates cardioprotection during ischemia reperfusion in mice. PLoS ONE.

